# IgG4‐Related Lung Disease in a Former Smoker: A Challenging Diagnosis

**DOI:** 10.1002/rcr2.70538

**Published:** 2026-03-16

**Authors:** Serena Bellani, Federica Pezzuto, Davide Garbin, Beatrice Scapolan, Luisa Santomartino, Fiorella Calabrese, Elisabetta Balestro, Nicol Bernardinello

**Affiliations:** ^1^ Respiratory Disease Unit, Department of Cardiac, Thoracic, Vascular Sciences and Public Health University of Padova Padova Italy; ^2^ Department of Cardiac, Thoracic, Vascular Science, and Public Health University of Padova Padova Italy

**Keywords:** hematologic diseases, immunoglobulin G4‐related disease, interstitial lung disease, lung cancer, rituximab

## Abstract

Immunoglobulin G4‐related disease (IgG4‐RD) is a rare systemic fibroinflammatory condition that may affect virtually any organ, including the lungs. Thoracic involvement often occurs with non‐specific imaging findings and requires histopathological confirmation. We present the case of a 71‐year‐old man with a history of T‐cell large granular lymphocytic leukaemia and MGUS, who presented with smoking‐related interstitial lung disease on high‐resolution chest computed tomography (CT) and left lower lobe (LLL) consolidation. Positron emission tomography (PET)/CT revealed hypermetabolic activity in lung consolidations and mediastinal and diaphragmatic lymph nodes. A right diaphragmatic lymph node biopsy showed increased IgG4‐positive plasma cells (IgG4/IgG > 40%). Subsequent transbronchial biopsies confirmed the presence of dense lymphoplasmacytic infiltrates, fibrosclerosis and IgG4‐positive plasma cells in lung tissue. Given the patient's diabetes, rituximab (RTX) was chosen over corticosteroids. RTX, consisting of four weekly infusions, led to significant clinical improvement and complete metabolic response on follow‐up PET/CT. Reporting this case, we would like to emphasise the importance of considering IgG4‐RD in the differential diagnosis of atypical thoracic infiltrates and highlight the role of RTX as an effective therapeutic option in patients for whom corticosteroids are contraindicated.

## Graphical abstract

This case highlights the importance of a structured and comprehensive diagnostic approach for patients with complex clinical presentations. It also demonstrates the potential role of rituximab therapy in treating IgG4 disease.

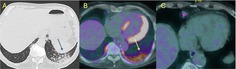

## 1 Introduction

Immunoglobulin G4‐related disease (IgG4‐RD) is a rare systemic fibroinflammatory condition characterised by a dense lymphoplasmacytic infiltrate, storiform fibrosis and obliterative phlebitis [1]. The disease can affect virtually any organ system, most commonly the pancreas, salivary and lacrimal glands, kidneys and, less frequently, the lungs [1]. Thoracic involvement has been increasingly recognised but remains a diagnostic challenge or a clinical challenge, as radiological findings are often non‐specific and may mimic infectious, inflammatory, or neoplastic processes; thus, histopathological confirmation is frequently required [2]. Pulmonary IgG4‐RD can present with mass‐forming lesions, nodules, or consolidations that may closely resemble primary lung carcinoma both clinically and radiologically. Given this overlap, awareness of this entity is essential to avoid misdiagnosis and unnecessary invasive procedures. Herein, we describe a rare case of IgG4‐RD with lung involvement, mimicking a lung carcinoma and successfully treated with Rituximab.

## 2 Case Report

A 71‐year‐old male, former smoker (28 pack‐year) with a family history of lung cancer, was referred to our clinic for further assessment. Since 2018, he had been under hematologic surveillance for T‐cell pseudolymphoma and T‐cell large granular lymphocytic leukaemia (T‐LGLL), confirmed by immunophenotyping (CD3^+^/CD8^+^/CD16^+^/CD57^+^) and identification of the STAT3 D661Y mutation. He was also diagnosed with monoclonal gammopathy of undetermined significance (MGUS), IgG kappa subtype. His past medical history included hypertension, type 2 diabetes, carotid atherosclerosis and benign prostatic hyperplasia (BPH) with urinary symptoms. In 2019, he underwent percutaneous coronary intervention (PCI) with a coronary stent.

**FIGURE 1:**
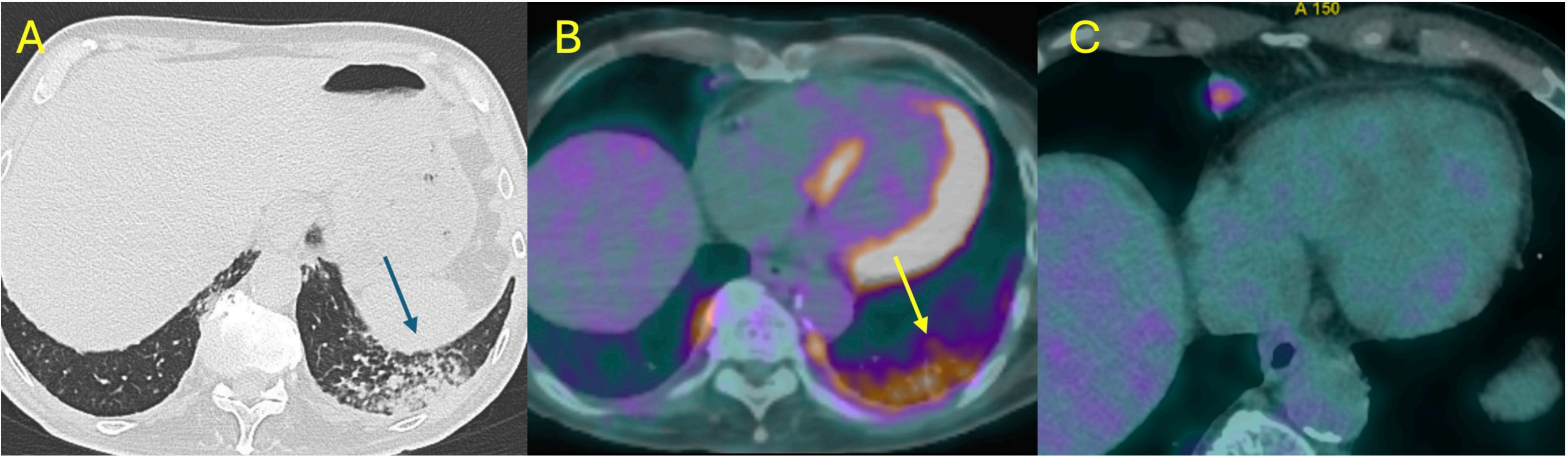
(A) CT scan demonstrated lung consolidation in the lower left lobe (blue arrow). (B) PET scan showed an intense captation in the lower left lobe (yellow arrow). (C) PET scan revealed a significant captation in a right diaphragmatic lymph node.

**FIGURE 2:**
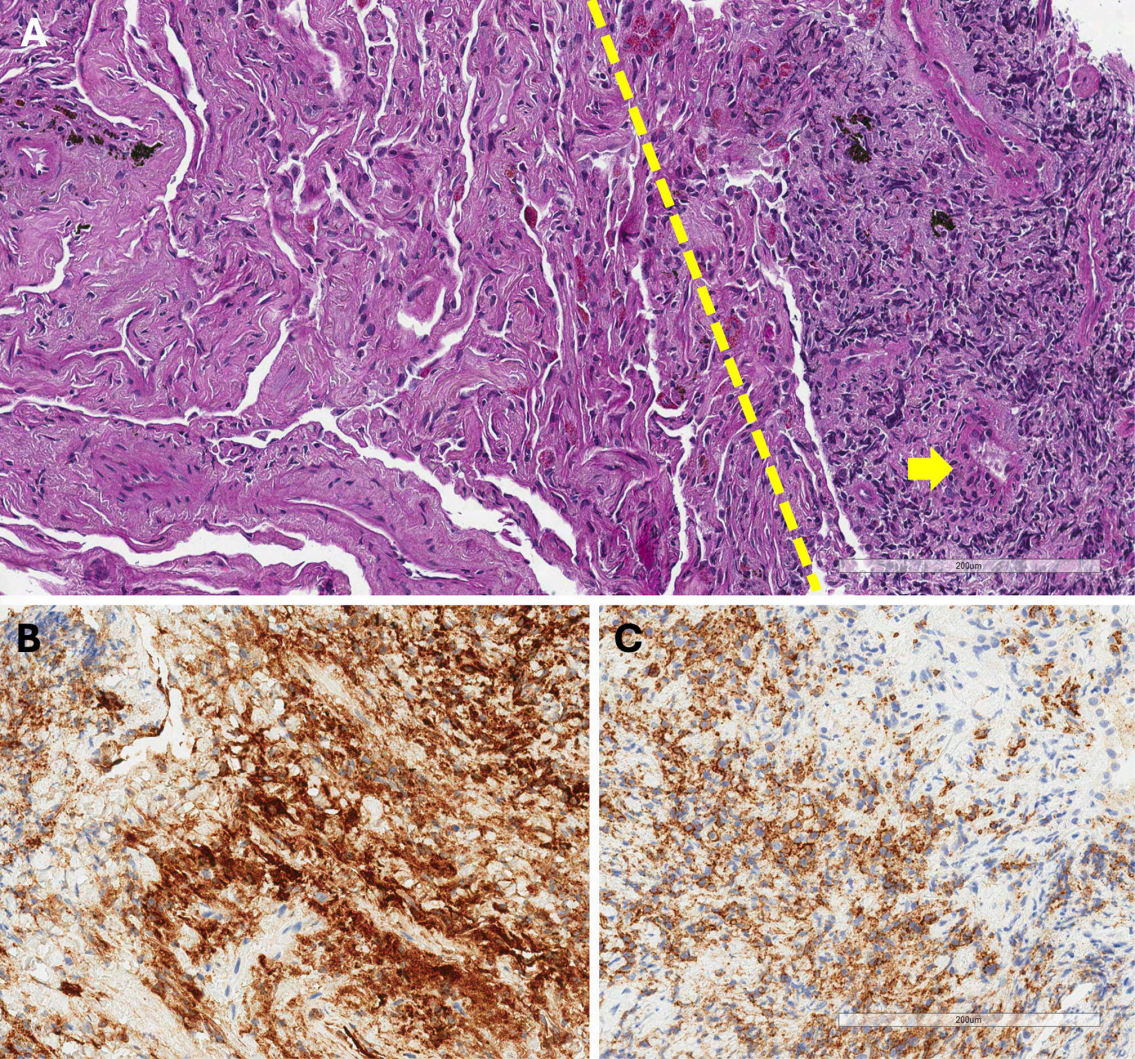
The biopsy showed a dense lymphoplasmacytic infiltrate with abundant plasma cells, associated with storiform fibrosis (left of the dotted line) and small‐vessel vasculitis (A, H&E, scale bar: 200 μm; bold yellow arrow). Immunohistochemistry demonstrated numerous IgG‐positive plasma cells and a significant subset of IgG4‐positive plasma cells, with an IgG4/IgG ratio > 40% (B, IgG immunostaining; C, IgG4 immunostaining; scale bar: 200 μm).

In August 2024, the patient was admitted to the Neurology ward for evaluation of a myopathy of uncertain aetiology. During hospitalisation, a CT scan revealed interstitial lung changes (ILDs), considered compatible with smoking‐related interstitial lung disease (ILD) after multidisciplinary discussion, along with left lower lobe (LLL) pulmonary consolidation (Figure 1) and multiple enlarged mediastinal lymph nodes. The patient denied any fever or cough. The only respiratory symptom reported was exertional dyspnea. To investigate a possible malignancy, a PET/CT scan was performed, showing mild uptake in LLL and hypermetabolic lymph nodes, most notably a right diaphragmatic node. Biopsy of this lymph node demonstrated a high number of IgG4‐positive plasma cells and an IgG4/IgG ratio > 40%, and markedly elevated serum IgG4 (11,900 g/L), raising suspicion for IgG4‐related disease (IgG4‐RD). Transbronchial biopsies and bronchoalveolar lavage were subsequently performed. Histology confirmed IgG4 plasma cell infiltration in the lung parenchyma with no evidence of infection. Immunophenotyping (CD38^+^) again showed an IgG4/IgG ratio > 40%, supporting a diagnosis of IgG4‐related inflammatory‐fibrotic lung disease (Figure 2). Serum IgG4 increased further to 17,530 g/L. Rituximab therapy was initiated (weekly with 4 weeks of duration). After 3 months, follow‐up PET/CT revealed a marked metabolic response, with complete resolution of prior abnormalities. This correlation with a significant reduction in serum IgG4 (3142 g/L) and significant clinical improvement.

## 3 Discussion

IgG4‐RD is an immune‐mediated condition characterised by fibroinflammatory lesions that can affect nearly any organ [1]. It may mimic malignancy, infection, or other immune‐mediated diseases [2]. Thoracic involvement demonstrates the greatest diversity of clinical and radiological presentation, requiring histological confirmation for diagnosis [2]. In 2019, ACR/EULAR proposed new classification criteria for IgG4‐RD, which rely on: (1) clinical‐radiological involvement of typical organs, or evidence of an inflammatory lymphoplasmacytic infiltrate in these organs; (2) exclusion of alternative diagnosis; and (3) a weighted scoring system incorporating clinical, serologic, radiologic and histopathologic findings. A total score ≥ 20 supports classification [3]. Other frameworks also exist, including the 2020 Revised Comprehensive Diagnostic criteria [4] and the original 2011 comprehensive criteria [5]. Comparative studies show differences in sensitivity and specificity, highlighting their different but complementary roles in clinical practice and research. Although these alternative criteria are widely used, the ACR/EULAR 2019 criteria were fully applicable to our patient.

Radiologically, pulmonary IgG4‐RD may present with nodular, ground‐glass, interstitial, or peribronchovascular changes or lesions. Other thoracic manifestations include pleural disease, mediastinal fibrosis and lymphadenopathy [3, 6]. In our case, despite the initial evidence of IgG4‐positive plasma cell infiltration in the right diaphragmatic lymph node, confirmation of organ involvement was required to meet diagnostic entry criteria for IgG4‐RD. Establishing the nature of the LLL lung consolidation was necessary both to assess pulmonary IgG4‐RD and to exclude other conditions, including malignancy or infection. In this context, all diagnostic inclusion criteria were strictly applied. The previously described histopathological findings, together with immunohistochemical evidence of an IgG4/IgG ratio > 40% and > 20 IgG4^+^ plasma cells per high‐power field with abundant CD38^+^ plasma cells, fulfilled the required histological component of the ACR/EULAR classification. Bronchoalveolar lavage further supported a lymphocytic inflammatory profile, showing lymphocytosis, reduced macrophages, normal granulocyte proportion, elevated CD4/CD8 ratio and normal NK and B cell percentages (Table 1).

**TABLE 1:** Application of the 2019 ACR/EULAR classification criteria for IgG4‐related disease.

The 2019 ACR/EULAR classification criteria for IgG4‐related disease	Patient evaluation	Numeric weight
Step 1. Entry criteria Characteristic clinical or radiologic involvement of a typical organ (e.g., pancreas, salivary glands, bile ducts, orbits, kidney, lung, aorta, retroperitoneum, pachymeninges, or thyroid gland [Riedel's thyroiditis]) **OR** pathologic evidence of an inflammatory process accompanied by a lymphoplasmacytic infiltrate of uncertain aetiology in one of these same organs	Yes	—
Step 2. Exclusion criteria: domains and items	No specific clinical, serological, pathological and Radiological findings	—
Step 3. Inclusion criteria	– *Histopathology*: dense lymphoplasmacytic infiltrate	+4
	– *Immunostaining*: IgG4/IgG > 40% and the number of IgG4+ cells/hpf is ≥ 10	+14
	– *Serum IgG 4 concentration* > 5× upper limit of normal	+11
	– *Chest*: Peribronchovascular and septal thickening	+4

*Note:* According to the 2019 ACR/EULAR classification criteria [3] for IgG4‐related disease, a minimum total score of 20 is required to establish the diagnosis. The patient fulfils the entry criteria, presents no exclusion criteria and achieves a cumulative inclusion score of 33 (4 + 14 + 11 + 4), thus exceeding the classification threshold. These findings support the diagnosis of IgG4‐related disease.

Therapeutic decisions in IgG4‐RD depend on clinical severity and the extent of organ involvement. While asymptomatic patients with limited disease may be monitored, symptomatic or progressive disease warrants treatment. First‐line therapy typically consists of corticosteroids at 0.6 mg/kg/day for 2–4 weeks with tapering. Rituximab is considered a second‐line agent with demonstrated efficacy [2]. Because the patient had diabetes mellitus and firmly refused steroid therapy, he was treated with intravenous rituximab, resulting in marked clinical and radiological improvement. Maintenance infusions are administered every 6 months, and the patient remains under regular controls.

An additional critical consideration in the diagnostic work‐up of pulmonary IgG4‐related disease is the exclusion of alternative conditions before initiating immunosuppressive therapy, particularly underlying lymphoproliferative disorders. In plasma cell‐rich or lymphoplasmacytic infiltrates, immunosuppression may transiently attenuate neoplastic components, potentially masking indolent haematological malignancies and delaying their recognition [7]. This issue is especially relevant given the documented histopathological overlap between IgG4‐related disease and low‐grade B‐cell lymphomas or plasma cell neoplasms, as well as with certain autoimmune or immunodeficiency‐related lung disorders [7]. Therefore, a comprehensive evaluation, including assessment of light‐chain restriction and, when appropriate, ancillary studies such as flow cytometry or molecular clonality analysis, is essential prior to treatment initiation [7].

Notably, as reported in the literature, an association may exist between the patient's T‐cell lymphoproliferative disorders and the development of IgG4‐RD. While some patients with underlying IgG4‐related disease later developed Non‐Hodgkin Lymphoma (NHL), it is also plausible that pre‐existing hematologic disease in our patient may have represented a predisposing factor for IgG4 dysregulation. Additionally, the patient's ILD could itself overlap with or mimic manifestations of pulmonary IgG4‐RD [6, 7, 8].

This case highlights the importance of a structured and comprehensive diagnostic approach in patients with complex clinical presentations. Despite confounding comorbidities, histological evaluation was necessary to establish a definitive diagnosis of pulmonary IgG4‐RD and to initiate appropriate targeted immunosuppressive therapy.

## Author Contributions

All authors are doctors of the University Hospital of Padova and members of the multidisciplinary team who took care of the patient and contributed to the case report writing and review.

## Funding

The authors have nothing to report.

## Consent

The authors declare that written informed consent was obtained for the publication of this manuscript and accompanying images using the consent form provided by the Journal.

## Conflicts of Interest

The authors declare no conflicts of interest.

## Data Availability

Data sharing not applicable to this article as no datasets were generated or analysed during the current study.
